# Analysis of the mRNA modification machinery alterations in breast cancer through the SCAN-B cohort

**DOI:** 10.1093/narcan/zcaf027

**Published:** 2025-09-03

**Authors:** Carlos Peula, Margalida Esteva-Socias, Kanchan Kumari, Erik Dassi, Francesca Aguilo

**Affiliations:** Department of Cellular, Computational and Integrative Biology (CIBIO), University of Trento, 38123 Trento, Italy; Department of Molecular Biology, Umeå University, 901 87 Umeå, Sweden; Wallenberg Centre for Molecular Medicine, Umeå University, 90187 Umeå, Sweden; Department of Molecular Biology, Umeå University, 901 87 Umeå, Sweden; Wallenberg Centre for Molecular Medicine, Umeå University, 90187 Umeå, Sweden; Department of Cellular, Computational and Integrative Biology (CIBIO), University of Trento, 38123 Trento, Italy; Department of Molecular Biology, Umeå University, 901 87 Umeå, Sweden; Wallenberg Centre for Molecular Medicine, Umeå University, 90187 Umeå, Sweden

## Abstract

Epitranscriptomic modifications regulate gene expression and have been implicated in cancer, including breast cancer. Using the SCAN-B cohort, we analyzed 49 messenger RNA modification regulators (mRMPs) across breast cancer subtypes. In the basal subtype, we found significant overexpression of m^6^A readers (IGF2BP1-3), m^5^C regulators (NSUN5, ALYREF, YBX1, YBX2), pseudouridine [PUS1, MARS (or MetRS), RPUSD2], and RNA editing enzymes [APOBEC3A (A3A), A3G, ADAR1], all linked to poor survival. Conversely, the m^6^A writer METTL14 was downregulated. Our findings highlight key mRMPs as potential biomarkers and therapeutic targets, underscoring the role of RNA modifications in breast cancer progression.

## Graphical Abstract

**Graphical Abstract:**
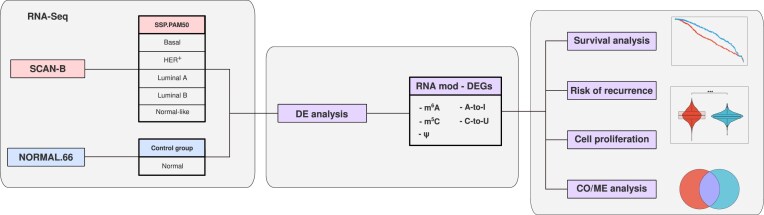

## Introduction

Epitranscriptomics is the study of chemical modifications of RNA molecules that regulate gene expression at transcriptional, post-transcriptional, and translational levels. To date, >170 distinct RNA modifications have been identified across various RNA species in all organisms, including over 60 modifications in eukaryotic RNA [1]. These chemical marks exhibit diverse functional roles depending on the RNA type and the specific location of the modification within the transcript. Although base modifications in ribosomal RNAs (rRNAs) and transfer RNAs (tRNAs) have long been recognized, the functional relevance of modifications in messenger RNAs (mRNAs) has only recently gained significant attention. These modifications have been shown to play critical roles in processes such as translation, RNA splicing, mRNA transport, and degradation, thereby affecting various biological processes [2–8]. Notably, dysregulation of mRNA modifications has been implicated in the pathophysiology of several cancer, including breast cancer [9–12].

Breast cancer is the most commonly diagnosed cancer among women worldwide and ranks as the second leading cause of cancer-related mortality in this group [13]. It is a highly heterogeneous disease, defined by distinct molecular subtypes with unique histopathological and biological features, which lead to varying responses to treatment. Five primary intrinsic breast cancer subtypes have been identified based on the expression levels of 50 specific genes, according to the PAM50 classification [14]. Luminal A/B tumors are estrogen receptor (ER) and progesterone receptor (PR) positive, with Luminal B showing higher expression of proliferation-related genes. The HER2 breast cancer subtype overexpresses the human epidermal growth factor receptor 2 (HER2). The basal subtype includes most of the triple-negative breast cancer (TNBC) cases, lacking the three conventional markers—ER, PR, and HER2. Normal-like tumors are less well characterized, share a similar immunohistochemical (IHC) pattern with the Luminal A subtype, and exhibit a normal breast tissue signature [15]. Among these subtypes, TNBC is associated with the worst prognosis, as hormonal therapies are ineffective. Therefore, a deeper understanding of the mechanisms driving TNBC is crucial.

In this study, we conducted a differential expression (DE) analysis of 49 messenger RNA modification regulators (mRMPs) (Supplementary Table S1), specifically *N^6^*-methyladenosine (m^6^A), 5-methylcytosine (m^5^C), pseudouridine, 2′-O-methylation, and RNA editing, using data from a large population-based cohort, the Swedish Cancerome Analysis Network—Breast (SCAN-B) initiative [16]. The integration of clinical, molecular, and genomic data in SCAN-B, along with longitudinal patient follow-up and comprehensive disease-stage representation, provides a unique opportunity to investigate mRNA modifications in breast cancer progression. Indeed, while other works have attempted the analysis of mRMPs in cancer for one or multiple modifications [12, 17], the unique features of this dataset are poised to provide more accurate and relevant results for the role of mRMPs in breast cancer. We assigned samples from SCAN-B to breast cancer subtypes using the single sample predictor classification method [18] and then identified mRMPs whose expression is dysregulated in specific subtypes. Focusing on the basal subtype, we observed that the m^6^A readers IGF2BP1, IGF2BP2, and IGF2BP3, the m^5^C methyltransferase NSUN5, and the m^5^C readers ALYREF, YBX2, and YBX1 were significantly overexpressed. Additionally, components of the pseudouridylation machinery, including the writers PUS1 and RPUSD2, as well as the reader MARS (also known as MetRS), along with RNA editing enzymes such as APOBEC3A (A3A), A3G, and ADAR1, were also upregulated. Most of these mRMPs are associated with poor survival. In contrast, the only downregulated mRMP we identified was the m^6^A writer METTL14. Furthermore, our analysis revealed the co-occurrence of alteration between regulators of m^5^C (NSUN5, ALYREF, YBX1, and YBX2) and Ψ (PUS1), hinting at a potential interplay between those modifications. Overall, our findings provide insights into the molecular landscape of breast cancer, highlighting key components of the RNA modification machinery that could serve as potential biomarkers for prognosis and therapeutic targets.

## Materials and methods

### SCAN-B data availability

The RNA-seq data corresponding to the SCAN-B samples and the 66 Normal cohort as well as the associated metadata used for this analysis can be found at https://data.mendeley.com/datasets/yzxtxn4nmd/4 [19].

### Selection of RNA modifiers with confirmed mRNA-binding function

In the present study, we considered a total of 49 mRMPs with confirmed binding to mRNA species among their pool of RNA targets, and representing six modification types (m^6^A, m^5^C, Ψ, 2′-O-Me, C-to-U, and A-to-I editing). The m^6^A interactors included the writer complex, namely METTL3, METTL14, RBM15, RBM15B, VIRMA, WTAP, ZC3H13, CBLL1, and the METTL16 writer. The m^6^A erasers (FTO and ALKBH5) and m^6^A readers (YTHDC1, YTHDC2, YTHDF1, YTHDF2, YTHDF3, IGF2BP1, IGF2BP2, IGF2BP3, HNRNPA2B1, HNRNPC, and FMR1) were also included.

For m^5^C, we included the known writers NSUN2, NSUN5, NSUN6, and NSUN7, along with the ALYREF reader and additional mRNA-binding proteins YBX1, YBX2, YBX3, and SRSF2, associated with m^5^C-mediated mRNA processing and stability. Additionally, TET1, TET2, and ALKBH1 were included as m^5^C erasers or regulators involved in m^5^C demethylation.

For Ψ, we considered the enzymes PUS1, PUS7, and TRUB1, along with DKC1 and RPUSD2, known to catalyze pseudouridylation in mRNA. MARS (metRS) was also included as Ψ reader due to its association with Ψ-modified transcripts. For 2′-O-Me, we selected FBL, a core component of the small nucleolar ribonucleoprotein (snoRNP) complex responsible for 2′-O-Me deposition, and CMTR1, a cap-specific 2′-O-methyltransferase involved in mRNA cap structure modifications. Regarding RNA editing, we included ADAR1 (or ADAR) and ADAR2 (or ADARB1), which catalyze the A-to-I editing, and the C-to-U editing enzymes APOBEC1, APOBEC3A, and APOBEC3G, as well as the cofactors RBM47 and A1CF.

The full list of RBPs can be found in Supplementary Table S1.

### Differential expression analysis for pairwise comparisons of breast cancer subtypes and against control in SCAN-B dataset

The differential expression analysis, comparing pairs of subtypes, was carried out with the DESeq2 R package (v1.40.2) [20]. Technical replicates of SCAN-B samples were collapsed according to the sample—merged association found in the metadata provided in [19]. Lowly expressed genes, with <10 counts in total, were excluded from the DE analysis.

Sequence count data were normalized using size factors (DESeq2’s median of ratios method) to adjust for the differences in sequencing depth and library size between samples. All breast cancer subtypes were contrasted against healthy breast tissue and the other subtypes for the DE analysis. The healthy samples consisted of 66 gene expression (GEX) profiles derived from normal breast tissue samples obtained from mammoplasty surgery from healthy women [18]. A threshold of adjusted *P*-value <.05 and |log_2_(FC)| > 1 was selected to define significantly up- and downregulated genes.

### Statistical analysis of RNA-seq data

Statistical analyses were conducted for mean pairwise comparisons between different breast cancer subtypes and conditions (i.e. Ki67 and ROR; high versus low). To this end, we used the two-tailed independent samples *t*-test with a threshold of *α* = 0.05. In the cases of multiple comparisons, *P*-values were adjusted using the Benjamini–Hochberg method. DESeq2-normalized gene counts were logarithmic transformed to center the median and avoid long-tailed boxplots.

### Computation of the Spearman correlation score for pairwise gene combinations

Correlations between RNA expression levels of different genes were evaluated for all RMPs. Spearman formula was applied to each pairwise gene comparison to assess positive or negative correlation. For each Spearman correlation, we set a threshold of *α* = 0.05. The *P*-values were adjusted for multiple testing using the Benjamini–Hochberg procedure.

### Dimensionality reduction and sample visualization

RNA-seq data for SCAN-B breast cancer samples were combined with the cohort of normal breast tissue samples for joint display. Principal component analysis (PCA) was performed for dimensionality reduction followed by 2D visualization of SCAN-B samples. The input gene count matrix was normalized using the variance stabilizing transformation prior to applying PCA.

### Survival analysis in the SCAN-B dataset

Evaluation of the prognostic effect of genes in breast cancer was carried out according to the overall survival (OS) time and events annotated in the SCAN-B supplementary table containing the samples metadata [19]. In parallel, we performed a second OS analysis of the METABRIC dataset for breast cancer available in the cBioPortal [21].

The OS time in days and the death status of the patient binned to 0/1 were employed to construct the Kaplan–Meier (KM) model with the survival R package (v3.5.7), while OS plots were generated with survminer (v0.5.0) [22, 23]. For each survival curve, samples were stratified according to gene expression. The 25th and 75th quantiles were selected to segregate gene expression into high (GEX > q75), intermediate (>q25 and ≤q75), or low (≤q25) samples. The False Discovery Rate (FDR) method was employed for multiple test correction of *P*-values obtained from the log-rank test.

The Cox proportional hazard model was employed to calculate the effect size (hazard ratio) for each KM curve. The adjustment for clinical covariates was conducted with the multivariable Cox model. In particular, the adjustment included the patient age, the Nottingham histological grade, and the treatment type.

The combined effect of basal-upregulated genes in OS was assessed by averaging their RNA expression levels. Samples expressing high RNA levels in the majority of genes were included in the high-expression group, whereas samples with the majority of genes lowly expressed constituted the low-expression group.

### Identification of CO and ME between up- and downregulation of mRMPs in basal samples

To assess the presence of alterations, we computed the fold change between the DESeq2-normalized counts of each basal subtype sample and the mean expression of normal samples. Only samples with |log_2_FC()| > 1 were considered as altered for each of the RMPs tested (upregulated, if log_2_FC > 1, and downregulated, if log_2_FC < −1), thus building a binary matrix of 1 and 0 representing altered and unaltered samples, respectively, for each factor.

The analysis of CO and ME was performed with SELECT v1.6 [24] and DISCOVER v0.9.4 [25]. Both tools evaluate pairwise alteration patterns by comparing observed data against a randomized null mode to determine statistical significance. The wMI *P*-values of SELECT were considered negative for ME mRMP pairs and positive for CO. Only interactions under the FDR cutoff and the average sum correction effect size threshold were considered significant.

### Gene set enrichment analysis of IGF2BP1–3 targeted genes

IGF2BP1–3 target genes were identified from eCLIP-derived RNA-binding sites of two cell lines (HepG2 and K562) from the ENCODE database [26]. Only IGF2BP1 had experimental data in both HepG2 and K562, while IGF2BP2 and IGF2BP3 data were exclusively available in K562 and HepG2, respectively. We thus considered the overlapping genes between the cell lines for IGF2BP1 and the pool of genes from either K562 or HepG2 for IGF2BP2 and IGF2BP3. Genes mapped to each RNA-binding site were annotated with CTK [27]. We employed clusterProfiler v. 4.10.0 [28] for the gene set enrichment analysis.

## Results

### *N^6^*-methyladenosine

m^6^A is the most abundant internal chemical modification of mRNA. It is catalyzed by METTL3 and METTL14, with METTL3 serving as the catalytic core of the m^6^A writer complex [29]. This complex also includes other components such as WTAP, VIRMA, CBLL1 (also referred to as HAKAI), ZC3H13, RBM15, and RBM15B. m^6^A is reversible, as it can be demethylated by the erasers FTO and ALKBH5. The readers of this mark, including YTHDF1–3, YTHDC1–2, IGF2BP1–3, HNRNPA2B1, HNRNPC, and FMR [30], influence the fate of the m^6^A-modified mRNA by modulating its splicing, stability, and translation [31–33].

**Figure 1.**
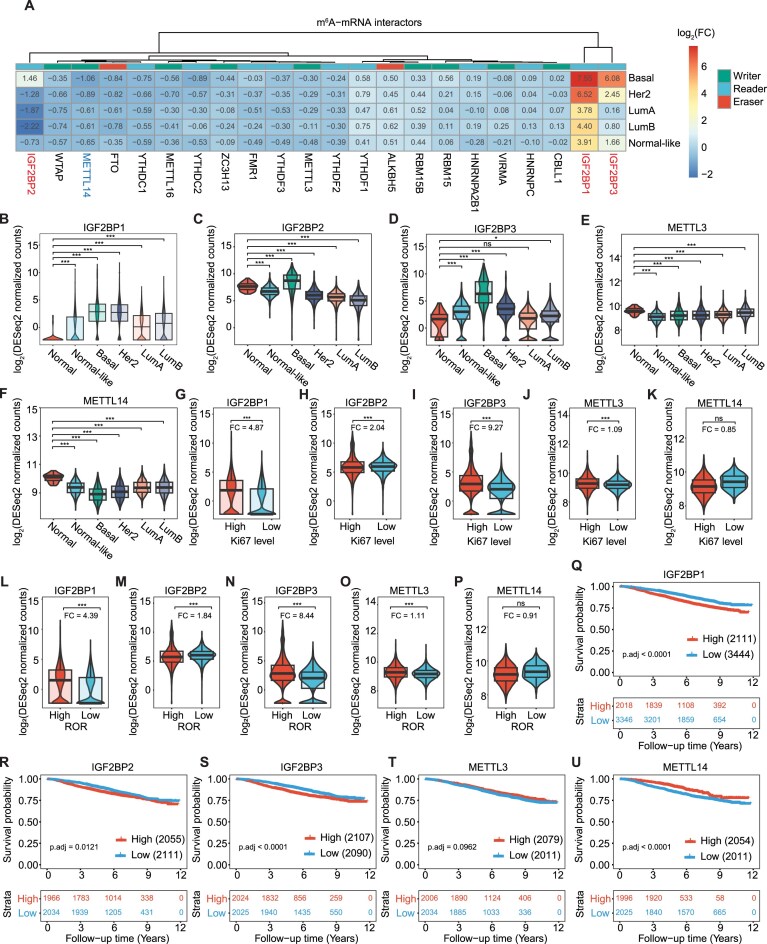
DE analysis of the m^6^A machinery revealed an upregulation of IGF2BP1/2/3 and downregulation of METTL14 in breast cancer. (**A**) The heatmap displays the log_2_(FC) values obtained from the DE analysis of the m^6^A regulators across breast cancer subtypes against the cohort of the normal breast samples (control group). Each row represents a different subtype versus control comparison, and each column is a different mRMP. Up- and downregulated RMPs in the basal subtype are highlighted in red and blue, respectively. (**B**–**F**) Boxplot set with gene expression (DESeq2-normalized gene counts) across the different breast cancer subtypes and the cohort of normal breast samples. RNA expression was measured as DESeq2-normalized counts. (**G**–**K**) Comparison of differentially expressed m^6^A-mRNA regulators between groups of high and low cell proliferation rates (Ki67 levels). (**L**–**P**) Comparison of RNA levels for m^6^A-mRNA regulators between groups of high and low risk of recurrence (ROR). (**Q**–**U**) KM curves constructed with stratified RNA expression of differentially expressed m^6^A-mRNA regulators (i.e. low if <q25 and high if >q75). The *x-*axis represents the time in years from the diagnosis to the last follow-up or death and *y-*axis is the survival probability.

In our study, we assessed the expression of the entire m^6^A machinery, including METTL16, an independent methyltransferase with high target specificity, primarily catalyzing the methylation of U6 spliceosomal RNAs (Supplementary Fig. S1A–E). A comparison of expression levels across breast cancer subtypes revealed an upregulation of the readers IGF2BP1, IGF2BP2, and IGF2BP3 in basal cases, whereas other m^6^A readers manifested a more homogeneous expression across subtypes (Fig. 1A–D and Supplementary Fig. S2C–H). While METTL3 and METTL14 have been often reported to be upregulated in breast cancer in previous studies [11, 34–39], METTL14 was found to be downregulated in the SCAN-B cohort, a trend that is also prevalently observed in The Cancer Genome Atlas (TCGA) cohort when METTL14 is altered [12]. Instead, METTL3 showed no biological significant changes (Fig. 1E and F). Additionally, the co-expression of genes across SCAN-B samples was assessed by computing the Spearman correlation coefficient for each pair of mRMPs. The results revealed moderately positive correlations between IGF2BP2 and 3, 1–2, and 1–3 (correlation scores: 0.4, 0.25, and 0.22, respectively). Moreover, we identified a highly correlated group of m^6^A regulators (scores ranging from 0.4 to 0.7), namely METTL14, YTHDC1, YTHDC2, and CBLL1, suggesting stronger co-expression patterns between these mRMPs (Supplementary Fig. S2I). Breast cancer cases with high proliferation rates exhibited elevated IGF2BP1, IGF2BP2, and IGF2BP3 expression (FC: 4.87, 2.04, and 9.27, respectively) (Fig. 1G–I), while no significant correlation was found between Ki67 expression and METTL14. Likewise, increased METTL3 levels were linked to greater proliferation, suggesting that specific breast cancer cases with elevated METTL3 expression tend to proliferate more (Fig. 1J and K). Moreover, gene set enrichment analysis revealed that IGF2BP1–3 target genes upregulated in the basal subtype are associated with several cancer-relevant biological processes, including focal adhesion, cell–substrate junction organization, cytoskeleton regulation, and mitotic nuclear division (Supplementary Fig. S3A and B, and Supplementary Table S2). Similarly, IGF2BP1–3 targets overexpressed in high Ki67 samples compared to low Ki67 samples were linked to enhanced proliferative activity. In particular, we observed significant enrichment of GO terms such as microtubule cytoskeleton organization, nuclear division, and mitotic cell cycle phase transition, among others (Supplementary Fig. S3C and D, and Supplementary Table S2).

Additionally, samples with a high ROR were associated with increased expression levels of IGF2BP1, IGF2BP3, and METTL3 (FC: 4.39, 8.44, and 1.11, respectively), whereas no such link was observed for IGF2BP2 or METTL14 (Fig. 1L–P). Poorer OS was correlated with high levels of the IGF2BPs and low METTL14 expression, while METTL3 expression showed no clear association with OS (Fig. 1Q–U). Analysis of other publicly available breast cancer dataset from TCGA and the Molecular Taxonomy of Breast Cancer International Consortium (METABRIC) revealed similar trends in OS, though the findings were not statistically significant (Supplementary Fig. S2J–N). In summary, we identified distinct expression patterns and coregulation of m^6^A machinery components in the basal subtype, suggesting the prognostic relevance of m^6^A regulation in breast cancer.

### 5-Methylcytosine

m^5^C is well characterized in tRNAs and rRNAs, but its functional significance in mRNAs has only recently gained attention. This modification is catalyzed by RNA methyltransferases of the NOL1/NOP2/SUN domain family (NSUN1–7), with NSUN2 and NSUN6 acting as the primary enzymes that methylate specific cytosine internal residues within the mRNA, while NSUN5 can also contribute to mRNA methylation [40–43]. Notably, m^5^C is a reversible modification, as it can be oxidized and removed by the ten-eleven translocation 2 (TET2) dioxygenase [44]. Its functional impact is mediated by reader proteins such as ALYREF, which promotes mRNA export, and YBX1, which regulates mRNA stability and translation [45–47]. Recent findings also indicate that YBX2, a homolog of YBX1, acts as an m^5^C reader and mediates liquid–liquid phase separation [48].

**Figure 2.**
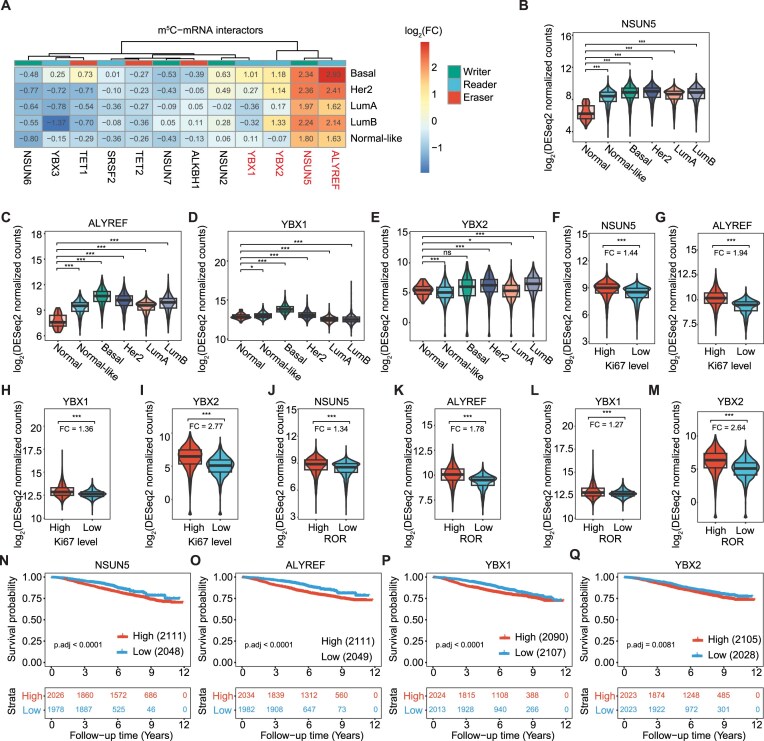
Upregulation of the m^5^C-mRNA interactors YBX1/2, NSUN5, and ALYREF in breast cancer. (**A**) The log_2_(FC) heatmap of m^5^C-mRNA regulators across breast cancer subtypes versus the control group. Each row corresponds to a different comparison, while each column represents distinct mRMPs. Significantly upregulated mRMPs are highlighted in red. (**B**–**E**) RNA levels of m^5^C mRMPs across the different breast cancer subtypes. (**F**–**I**) Comparison of overexpressed m^5^C-mRNA regulators between groups of high and low cell proliferation rates (Ki67 levels). (**J**–**M**) Comparison of differentially expressed m^5^C-mRNA regulators between groups of high and low ROR. (**N**–**Q**) KM curves constructed with stratified RNA expression of upregulated m^5^C mRMPs (i.e. low if <q25 and high if >q75). The *x-*axis represents the time in years from the diagnosis to the last follow-up or death and *y-*axis is the survival probability.

Comparison of the expression patterns of the m^5^C machinery across breast cancer subtypes revealed that the m^5^C methyltransferase NSUN5 and the readers ALYREF, YBX1, and YBX2 were overexpressed in the basal subtype relative to normal tissue (Fig. 2A and Supplementary Fig. S4A–M). Furthermore, the Spearman correlations for the m^5^C interactors NSUN5 and ALYREF are the ones with the highest score in the full SCAN-B cohort (0.77) (Supplementary Fig. S4N).

Basal cases characterized by high NSUN5, ALYREF, YBX1, and YBX2 expression (Fig 2B–E) exhibited enhanced proliferation rates (FC: 1.44, 1.94, 1.36, 2.77, respectively; Fig. 2F–I) and were associated with a heightened ROR (1.34, 1.78, 1.27, 2.64, respectively; Fig. 2J–M). Worse OS in the SCAN-B and METABRIC datasets was associated with elevated NSUN5, YBX1, and YBX2 expression (OR: 3.03, 1.63, and 1.5, respectively), while ALYREF levels were only associated with worse survival in SCAN-B (Fig. 2N–Q and Supplementary Fig. S5A–D). Altogether, our analysis highlights the potential oncogenic role of NSUN5, ALYREF, YBX1, and YBX2 in breast tumorigenesis.

### Pseudouridine

Pseudouridylation (Ψ) is another widespread RNA modification found in mRNA, tRNA, rRNA, and some small RNA species [49, 50]. It is catalyzed by two types of enzymes: stand-alone pseudouridine synthases (PUSs) and H/ACA small nucleolar ribonucleoprotein complexes [51, 52], which primarily mediate rRNA pseudouridylation. Some orphan snoRNAs and stand-alone PUSs, such as TRUB1, PUS7, RPUSD2, and PUS1, target mRNAs, influencing RNA stability, translation efficiency, and splicing [49, 53–58]. Methionine aminoacyl tRNA^Met^ synthetase (MetRS or MARS) acts as a reader protein for pseudouridylation that when binding to mRNA, confers gene-specific translation regulation [59]. This modification is irreversible and no eraser protein has been found up to now.

**Figure 3.**
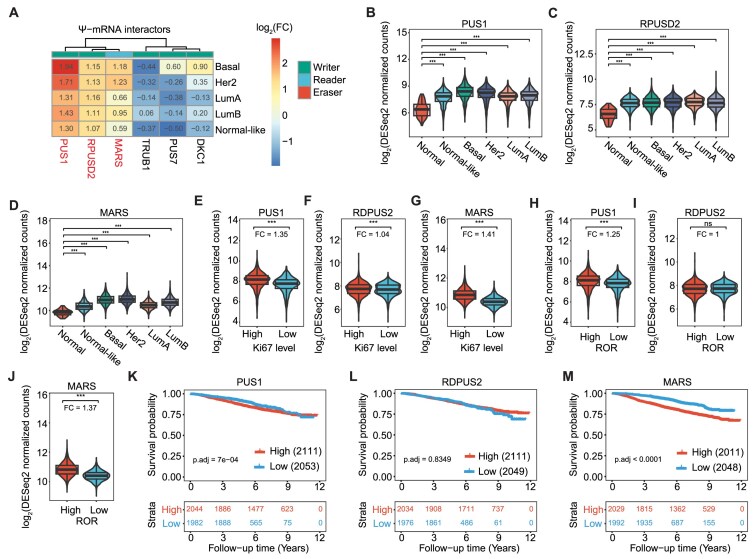
Upregulation of Ψ writers PUS1 and RPUSD2, and the Ψ reader MARS in breast cancer. (**A**) Heatmap displaying the log_2_(FC) of Ψ mRMPs for each breast cancer subtype-control comparison. Each row corresponds to a different comparison, while each column represents distinct mRMPs. Significantly upregulated mRMPs are highlighted in red. (**B**–**D**) RNA levels of Ψ mRMPs across the different breast cancer subtypes. (**E**–**G**) Comparison of overexpressed Ψ mRMPs between sample groups of high and low cell proliferation rates (Ki67 levels). (**H**–**J**) Comparison of differentially expressed Ψ-mRNA regulators between groups of high and low ROR. (**K**–**M**) KM curves constructed with stratified RNA expression of upregulated Ψ mRMPs (i.e. low if <q25 and high if >q75). The *x-*axis represents the time in years from the diagnosis to the last follow-up or death and *y-*axis is the survival probability.

We assessed the expression of mRMPs involved in the pseudouridylation of mRNA in the SCAN-B cohort, and found that the expression of PUS1, RPUSD2, and MARS was upregulated in the basal subtype compared to normal breast tissues (Fig. 3A–D and Supplementary Fig. S6A–F). Furthermore, we observed a positive correlation between RNA expression levels of these three mRMPs across SCAN-B samples (correlation range: 0.4–0.65; Supplementary Fig. S6G). The elevated expression of PUS1, RPUSD2, and MARS in breast cancer cases showed a positive correlation with high Ki67 levels (FC: 1.35, 1.04, and 1.41, respectively; Fig. 3E–G), suggesting their contributing role in tumor growth. We also observed that high ROR cases were associated with elevated PUS1 and MARS expression (FC: 1.25 and 1.37, respectively) (Fig. 3H–J). Notably, higher expression of PUS1 and MARS was significantly linked to reduced overall patient survival, while RPUSD2 expression showed no significant correlation with patient outcomes (Fig. 3K–M). Consistent with SCAN-B cohort findings, METABRIC analysis showed that high expression of PUS1 and MARS was linked to reduced overall patient survival (OR: 1.866 and 1.968) (Supplementary Fig. S6H–J). Although elevated RPUSD2 expression suggested a trend toward reduced survival, the results were not statistically significant. Altogether, these findings suggest a strong association of PUS1, MARS, and RPUSD2 with an oncogenic phenotype.

### RNA editing

RNA editing consists in site-specific base substitutions and insertion/deletions (indels) within the RNA sequence, excluding changes due to splicing and polyadenylation [60–62]. Two families of enzymes mediate single-nucleotide substitutions: ADARs (adenosine deaminases acting on RNA), which convert adenosine to the guanosine analog inosine (A-to-I), and AID/APOBECs (activation induced deaminase/apolipoprotein B mRNA editing enzyme, catalytic polypeptide), which catalyze cytosine-to-uracil (C-to-U) substitutions [60, 61, 63]. A-to-I editing in double stranded RNA (dsRNA) represents the most common type of editing in mammalian cells and is catalyzed by ADAR1 (or ADAR) and ADAR2 (or ADARB1) [64]. APOBEC enzymes possess a diverse range of functions that impact human health and diseases [65]. APOBEC3 (A3), the most diversified C-to-U enzyme family in humans, consists of seven proteins (APOBEC3A (A3A), A3B, A3C, A3D, A3F, A3G, and A3H) [66].

**Figure 4.**
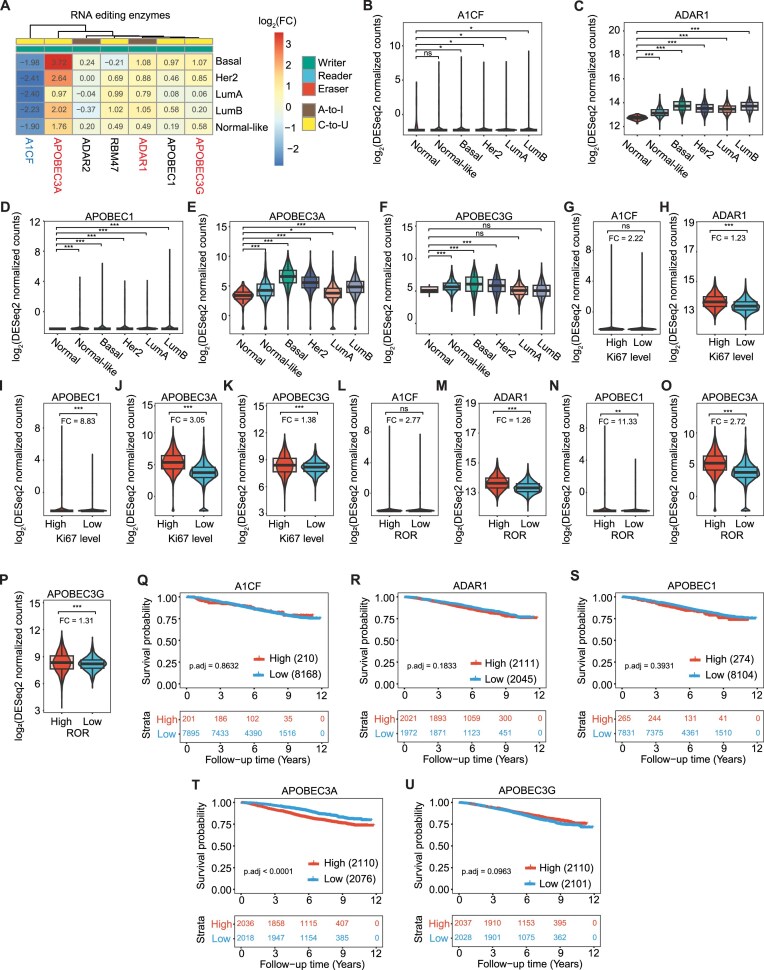
DE analysis of mRNA editing enzymes and cofactors in breast cancer. (**A**) log_2_(FC) heatmap of the mRNA editing enzymes and cofactors for each breast cancer subtypes against the cohort of the normal breast samples (control group). Each row represents a different subtype versus control comparison and each column is a different enzyme or cofactor. A-to-I or C-to-U activity is indicated by brown or yellow colors, respectively. Up- and downregulated enzymes are highlighted in red and blue, respectively. (**B**–**F**) RNA levels of mRNA-editing enzymes across the different breast cancer subtypes. (**G**–**K**) Comparison of differentially expressed mRNA-editing enzymes between sample groups of high and low cell proliferation rates (Ki67 levels). (**L**–**P**) Comparison of differentially expressed mRNA-editing enzymes between groups of high and low ROR. (**Q**–**U**) KM curves constructed with stratified RNA expression of target mRNA-editing enzymes (i.e. low if <q25 and high if >q75). The *x-*axis represents the time in years from the diagnosis to the last follow-up or death and *y* axis is the survival probability.

Analysis of the RNA editing machinery revealed that A3A, A3G, and ADAR1 were upregulated in the basal subtype compared to normal tissues (Fig. 4A–F and Supplementary Fig. S7A–H). Consistent with other mRMPs, we observed a moderate positive correlation in RNA expression levels among A3A, A3G, and ADAR1 (score range: 0.15–0.5) (Supplementary Fig. S7I). Notably, samples with high proliferation rates exhibited significantly elevated expression of ADAR1, A3A and A3G (FC: 1.23, 3.05, and 1.38, respectively) (Fig. 4G–K). Likewise, patients at high ROR showed increased expression of ADAR1, A3A, and A3G (FC 1.26, 2.72, and 1.31, respectively) (Fig. 4L–P). Furthermore, the OS analysis revealed a potential association between high expression of A3A and a poorer prognosis in the SCAN-B cohort (Odds ratio (OR): 1.723) (Fig. 4Q–U). In contrast, neither A3A or A3G manifested any significant effect in survival probability in the METABRIC dataset; in this case, only ADAR1 showed a significant negative impact on prognosis (OR: 1.68) (Supplementary Fig. S7J–N). In summary, our findings on SCAN-B are consistent with previous studies that described the strong association between dysregulated expression of RNA-editing enzymes and BC onset and progression [67–69].

### Potential interplay between different RNA modifications

**Figure 5.**
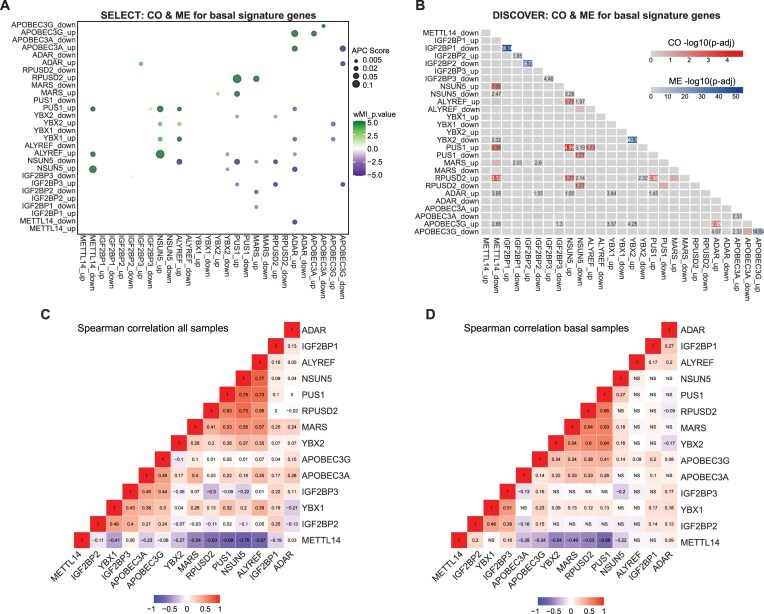
Interactions between RNA modifications. (**A**) Analysis of CO and ME between mRMPs employing SELECT algorithm. The color gradient represents the weighted mutual information (wMI) *P*-value, which indicates the type of interaction, ranging from ME (violet) to CO (green), whereas the bubble size represents the average sum correction (APC) effect size. (**B**) Heatmap of significant CO/ME interactions between up- and downregulated mRMPs, according to the DISCOVER tool results. Significant CO and ME are represented in red or blue, respectively. (**C**, **D**) Spearman correlation scores between mRMPs overexpressed in the basal subtype. The correlation was computed for the whole SCAN-B cohort (left matrix) and the basal subset samples of *n* = 734 (right matrix). The color gradient ranges represent the degree of correlation and range from −1 (blue) to +1 (red).

Emerging evidence suggests that different RNA modifications might interact, with the presence of one modification potentially facilitating or hindering the deposition of another [70]. To explore this aspect in breast cancer, we assessed co-occurrence and mutual exclusivity of mRMP alterations using two complementary tools: SELECT and DISCOVER. The resulting interaction matrices revealed significant co-occurrence for m^5^C-associated mRMPs and Ψ writers within the basal subtype. In particular, alterations of NSUN5, ALYREF, YBX1, and YBX2 were significantly co-occurrent. From those, NSUN5 and ALYREF were also co-occurrent with PUS1 (Fig. 5A). Additionally, NSUN5 downregulation was mutually exclusive with ALYREF and PUS1 upregulation, potentially suggesting different regulatory programs associated with different modification deposition levels. These associations were consistently identified by both tools, supporting their robustness. SELECT also identified a co-occurrence among ADAR1, A3A, and A3G, though this association was not reported by DISCOVER (Fig. 5A and B). In addition, the patterns of co-occurrence previously observed persisted when the full SCAN-B cohort was considered (Supplementary Fig. S8).

In parallel to this analysis, we also evaluated correlation among mRMPs overexpressed in the basal subtype using pairwise Spearman analysis, both across the SCAN-B cohort and within the basal subtype. Overall, the correlation patterns observed within the basal subset of samples resembled those of the full SCAN-B cohort (Fig. 5C and D). The resulting patterns were largely consistent, with positive correlations between m^5^C regulators and PUS1 mirroring the alteration co-occurrence findings and supporting potential regulatory interplay between m^5^C and Ψ modifications.

## Discussion

**Figure 6.**
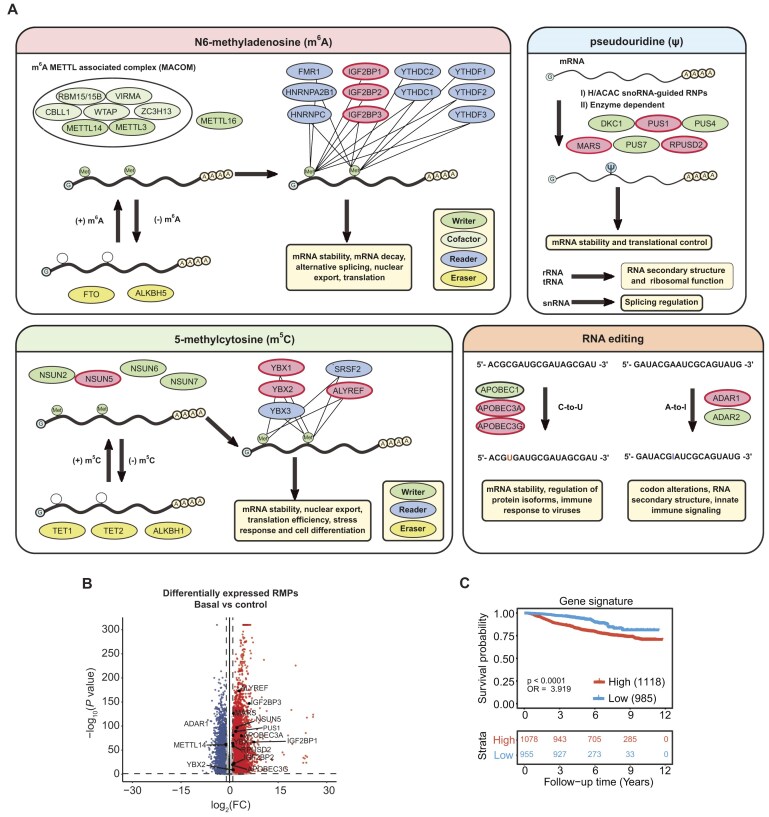
mRMP dysregulation and prognostic relevance in the basal subtype. (**A**) Schematic representation of mRNA modifications. mRMPs that are upregulated in basal samples are highlighted in red. (**B**) Volcano plot showing differentially expressed genes for the basal subtype versus control. The statistical significance of differentially expressed genes was determined employing the Wald test. Cutoffs for log_2_(*P*-val), and log_2_(FC) were set to 1.301 and 1, respectively. RMPs over the threshold of significance were labeled with their names. (**C**) OS analysis of the basal gene signature. KM curves constructed with the average score of high and low RNA expression levels of basal-overexpressed mRMPs. The *x-*axis represents the time in years from the diagnosis to the last follow-up or death and *y-*axis is the survival probability.

In this study, we identified mRMPs associated with clinicopathological features in breast cancer using the SCAN-B cohort (Fig. 6A). Specifically, we found that IGF2BP1, IGF2BP2, IGF2BP3, NSUN5, ALYREF, YBX1, YBX2, PUS1, RPUSD2, and MARS, along with RNA editing regulators enzymes such as APOBEC3A, A3G, and ADAR1, are upregulated in basal cases and serve as indicators of poor survival (Fig. 6B and C). In contrast, METTL14 was the only mRMP found to be downregulated in the basal subtype compared to normal tissues (Fig. 6B).

Dysregulated m^6^A regulators are linked to breast cancer [10]. METTL3 is widely recognized as a proto-oncogene, and elevated m^6^A levels in peripheral blood have been proposed as a potential diagnostic biomarker for breast cancer [71, 72]. However, in the SCAN-B cohort, METTL3 expression showed no significant association with the basal subtype. Notably, when upregulated, METTL3 was linked to increased proliferation and a higher ROR. Our study found that METTL14 is downregulated in the basal subtype, and its expression correlates with better clinical outcomes, consistent with previous findings [73, 74]. Conversely, other studies suggest that METTL14 enhances breast tumorigenesis by promoting m^6^A deposition on oncogene transcripts [75]. This highlights the complex, debated roles of m^6^A interactors across breast cancer subtypes.

IGF2BP1, IGF2BP2, and IGF2BP3 were upregulated in the SCAN-B cohort, aligning with their widespread dysregulation in cancer. IGF2BPs are among the most upregulated RNA-binding proteins in TCGA datasets and play key roles in carcinogenesis, often correlating with poor prognosis and tumor progression [76, 77]. These mRMPs bind m^6^A-marked mRNAs, enhancing their stability [76, 78]. The overexpression of IGF2BPs in the basal subtype suggests a shift toward mRNA stabilization over degradation, disrupting mRNA turnover. Our findings align with studies in other cancer types showing that IGF2BPs stabilize oncogenic mRNAs, such as *MYC* and *SRF* [79, 80]. As previously shown, IGF2BP1–3 also targeted other genes that participate in several mechanisms associated with an increased cell proliferation rate, potentially promoting cancer progression (Supplementary Fig. S3A–D). Similarly, tumor cells may promote oncogenic expression through m^5^C, as suggested by the upregulation of its readers, YBX1, YBX2, and ALYREF, which enhance mRNA transport, stability, and translation. Abnormal expression of YBX1 and ALYREF, and the m^5^C writer NSUN5 has been linked to poor survival, though the role of m^5^C in these cases remains underexplored. Additionally, since NSUN5 can modify both mRNA and rRNA, linking specific phenotypic effects to individual RNA modifications is challenging, further complicating functional interpretations.

Elevated pseudouridine levels have been correlated with advanced cancer stages and poor therapeutic response, with increased levels detected in biological samples from various malignancies [81–84]. Dysregulated expression of PUS enzymes has been linked to tumorigenesis and cancer progression. Depletion of PUS1 and MARS1 has been shown to suppress cancerous phenotypes in hepatocellular carcinoma, renal cell carcinoma, and breast cancer [84–86]. However, the role of RPUSD2 in cancer progression remains unexplored. Consistent with findings in breast and colorectal cancer, we observed increased expression of PUS1, MARS, and RPUSD2 in the SCAN-B cohort [87, 88]. These results suggest a potential role for PUS1-, MARS-, and RPUSD2-mediated pseudouridine modifications in breast cancer pathogenesis, warranting further investigation.

RNA editing, particularly A-to-I editing mediated by ADAR enzymes, has also been implicated in cancer. ADAR1 and ADAR2 dysregulation contributes to tumorigenesis [64, 89]. A-to-I editing can alter splicing regulatory elements, leading to aberrant splicing in numerous cancers, while also affecting mRNA stability, localization, translation, and interactions with RNA-binding proteins, ultimately impacting cancer progression [89–91]. Consistently with previous studies, ADAR1 was overexpressed in the basal subtype and high ADAR1 levels were associated with worse clinical outcomes [92]. Additionally, APOBEC enzymes, particularly APOBEC3A, have been implicated in cancer progression by inducing mutations that drive genome instability and tumor heterogeneity [60, 93]. Abnormal A3A expression has been reported in breast cancer, where it correlates with higher levels of APOBEC-signature mutations [94].

Although only two mRMPs, FBL and CMTR1, mediate 2′-O-methylation on mRNA, both were slightly upregulated in the basal subtype compared to normal tissues, but this upregulation was not significant (Supplementary Fig. S9A–C). However, their slight upregulation suggests a potential involvement in breast tumorigenesis as previously suggested (Supplementary Fig. S9B and C) [95]. High CMRT1 was associated with worse prognosis, while FBL showed no association with OS (Supplementary Fig. S9D–G). Notably, FBL is generally a marker of poor prognosis in early-stage breast cancer, though a subset of aggressive tumors (∼10%) show reduced expression [96]. Given its role in methylating both rRNA and mRNA, distinguishing its specific impact on mRNA remains challenging.

We acknowledge several limitations in our study. Gene expression was assessed solely at the RNA level in SCAN-B samples, which, while indicative of protein expression, does not provide direct proof. Protein expression analysis in this dataset would be essential to strengthen our findings. Additionally, while our analysis reveals significant associations, correlation does not imply causation. Further validation is required to determine whether these relationships reflect direct regulatory interactions or are influenced by confounding factors. Furthermore, several genes were significantly associated with worse prognosis in the univariable analysis. However, after adjusting for age, treatment, and histological grade in multivariable models, many of these associations were reduced, suggesting a partial confounding by these clinical factors (Supplementary Fig. S10A–C and Supplementary Table S3).

The co-occurrence observed between NSUN5, ALYREF, YBX1, YBX2, and PUS1 might suggest the existence of a common regulatory block involving m^5^C and Ψ mRMPs, upregulated in the basal subtype. Both RNA modifications are strongly associated with translation efficiency, and their deposition in tRNAs and rRNAs is well documented. However, a direct interaction between m^5^C and Ψ in mRNAs has not been well explored yet [97, 98]. Therefore, the coregulation observed for m^5^C and Ψ is probably not limited to mRNA regulation but rather reflects enhanced pathways involved in translation. In this context, our work raises the possibility of an enhanced m^5^C and Ψ deposition in the basal subtype with consequences in the translation rate of cancer cells.

Despite advances in experimental techniques, tools for detecting multiple RNA modifications simultaneously are still lacking, limiting our understanding of their interplay in cancer progression. While our work suggests potential crosstalks between different RNA modification machineries, direct measurement of the RNA modification status was not addressed. Therefore, differences in the abundance and localization of such modifications could not be evaluated. Moreover, some RNA-modifying enzymes can moonlight as proteins with unrelated roles, making it difficult to separate their canonical RNA-modifying activities from secondary functions. This complexity is further exemplified by METTL3, which has been shown to promote oncogene translation independently of its enzymatic function [8].

In conclusion, our study identifies novel mRMPs potentially involved in breast tumorigenesis, offering new insights into the molecular mechanisms of the disease. By highlighting these underexplored mRMPs, our findings pave the way for future research that could enhance breast cancer diagnostics and treatment, underscoring the critical role of RNA modifications in breast tumorigenesis.

## Supplementary Material

zcaf027_Supplemental_Files

## Data Availability

The RNA-seq data corresponding to the SCAN-B samples and the 66 Normal cohort as well as the associated metadata used for this analysis can be found at https://data.mendeley.com/datasets/yzxtxn4nmd/3 [19].
