# Supplementary material for: Analysis of the mRNA modification machinery alterations in breast cancer through the SCAN-B cohort
Source: NAR Cancer. 2025 Sep 3;7(3):zcaf027. doi: 10.1093/narcan/zcaf027 (PMC12409412; doi:10.1093/narcan/zcaf027)
Supplement: zcaf027_Supplemental_Files [file zcaf027_supplemental_files.zip › corrected_Extended_data_Peula_et_al.docx]

**EXTENDED DATA**

**Supplementary** **Table 1**. List of mRMPs analysed in this study.

**Supplementary Table 2.** IGF2BP targets upregulated in Basal vs control and high Ki67 vs low Ki67.

**Supplementary Table 3.** Odds ratios from overall survival analyses. Results from Cox Proportional risk model and Cox multivariate model.

**Supplementary Figure S1**. Principal Component Analysis (PCA) of SCAN-B samples and visualization of m^6^A regulators expressed across samples, related to Figure 1.

**Supplementary Figure S2**. PCA and Spearman correlation of m^6^A mRMPs in SCAN-B samples and OS analysis of METABRIC, related to Figure 1.

**Supplementary Figure S3**. Gene set enrichment analysis of IGF2BP targets.

**Supplementary Figure S4**. PCA and Spearman correlation of the m^5^C mRMPs across SCAN-B samples, related to Figure 2.

**Supplementary Figure S5**. OS analysis of TNBC-overexpressed m^5^C mRMPs in METABRIC, related to Figure 2.

**Supplementary Figure S6.** PCA and Spearman correlation for Ψ-related mRMPs across SCAN-B cohort and OS analysis of METABRIC, related to Figure 3.

**Supplementary Figure S7**. PCA and Spearman correlation for RNA editing enzymes in SCAN-B cohort and OS analysis of METABRIC, related to Figure 4.

**Supplementary Figure S8**. CO/ME analysis for all mRMPs across SCAN-B cohort, related to Figure 5.

**Supplementary Figure S9**. Analysis of Fibrillarin and CMRT1 in the SCAN-B dataset.

**Supplementary Figure S10**. Overall survival analyses for relevant clinical variables of SCAN-B samples.

**Figure S1**


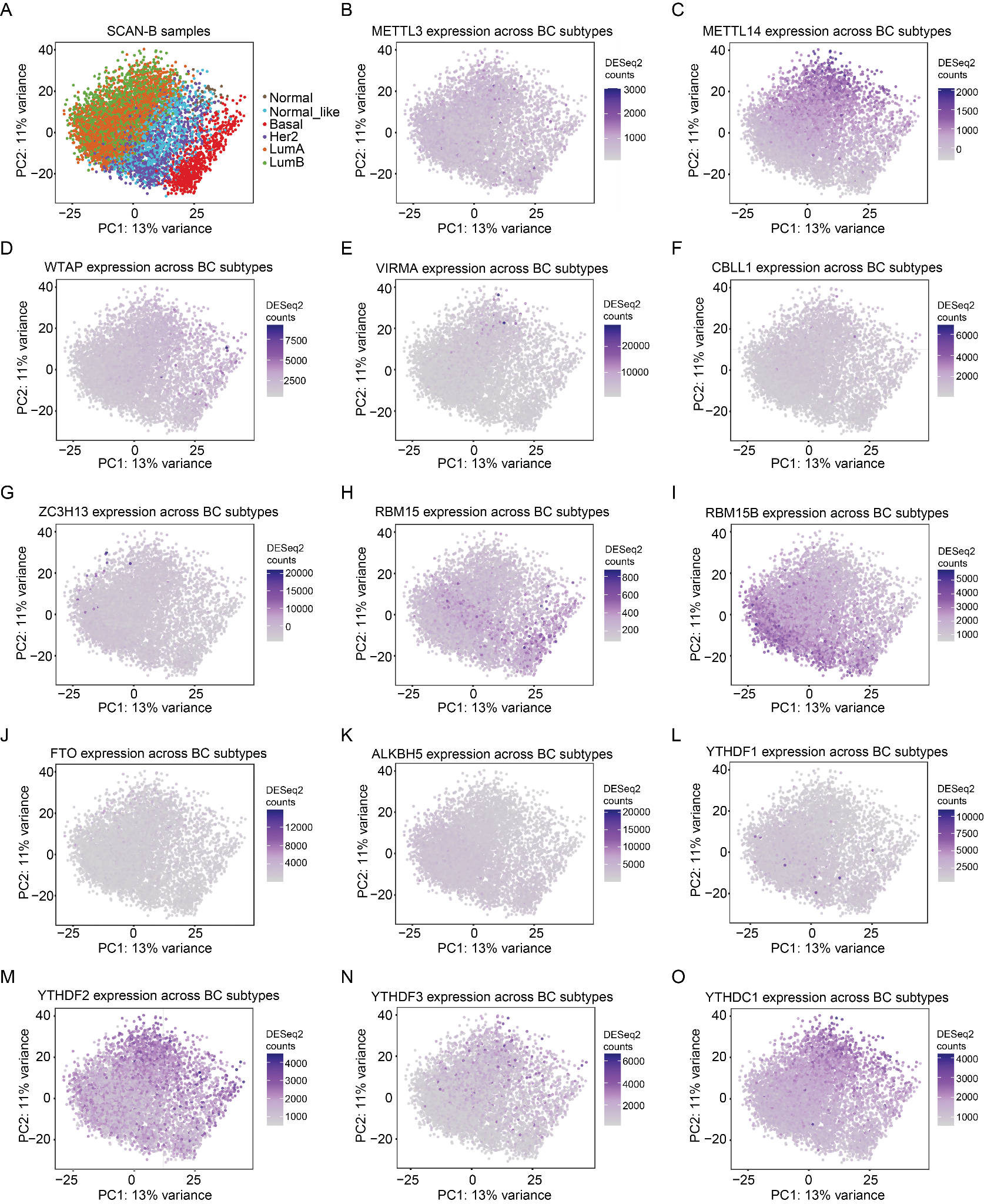


*Figure legend in the next page.*

**Supplementary Figure S1**. **Principal Component Analysis (PCA) of SCAN-B samples and visualization of m^6^A regulators expressed across samples, related to Figure 1.** (**A**) PCA with annotated subtypes. Each point on the scatter plot represents an individual sample. The color code corresponds to the breast cancer subtype. (**B-O**) Each plot corresponds to the gene expression of an m^6^A mRMP, where the color gradient reflects the RNA expression level across samples (DESeq2-normalized counts).

**Figure S2**


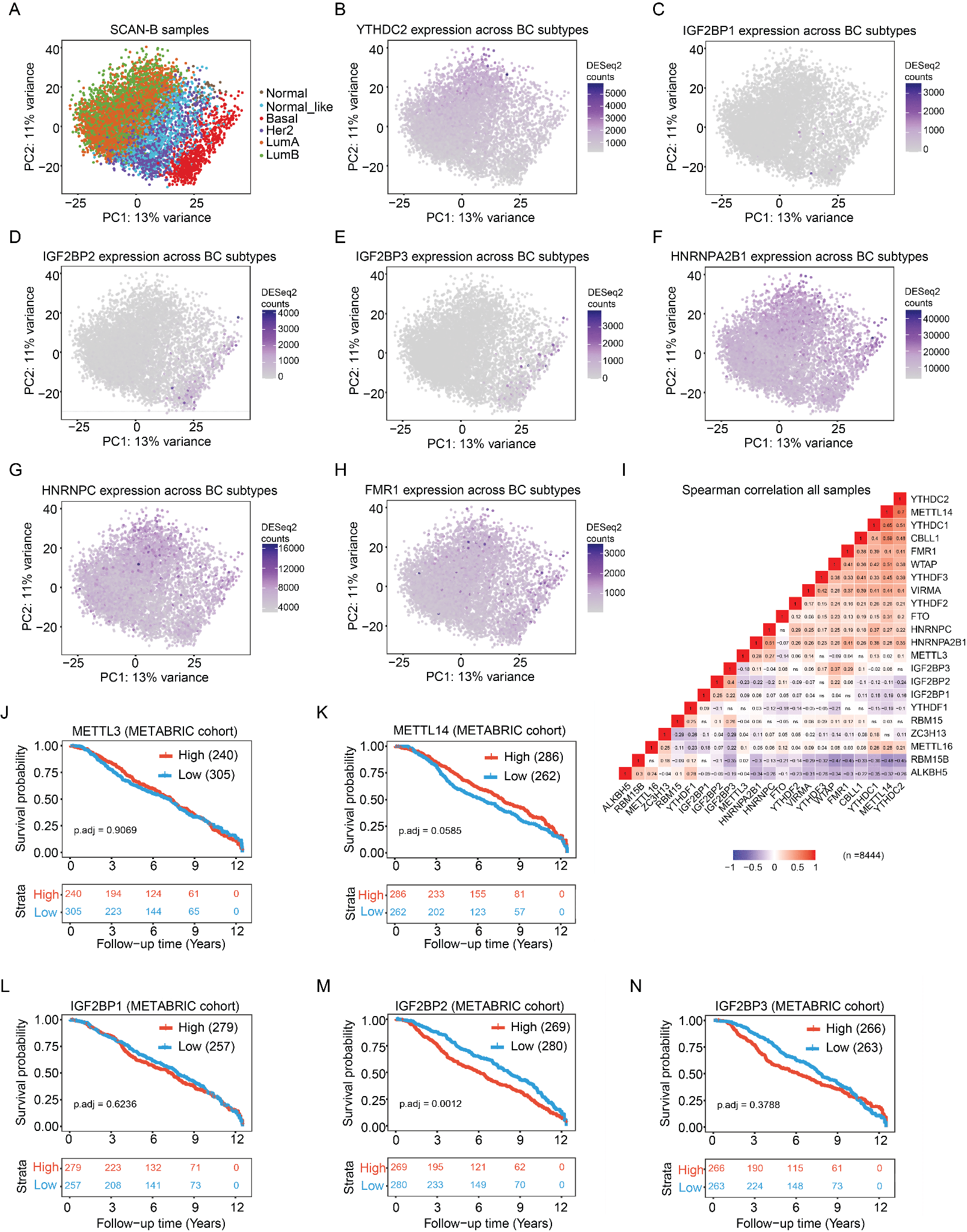


*Figure legend in the next page.*

**Supplementary Figure S2**. **PCA and Spearman correlation of m^6^A mRMPs in SCAN-B samples and OS analysis of METABRIC, related to Figure 1**. (**A**) PCA with annotated subtypes. Each point on the scatter plot represents an individual sample. The color code corresponds to the breast cancer subtype. (**B-H**) PCA plots showing gene expression of m^6^A mRMPs, where each point represents a SCAN-B sample and the color gradient reflects the RNA expression level across samples (DESeq2-normalized counts). (**I**) Spearman correlation scores between m^6^A mRMPs using the full SCAN-B cohort. The color gradient ranges represent the degree of correlation and range from -1 (blue) to +1 (red). (**J-N**) OS analysis conducted in the METABRIC dataset for m^6^A mRMPs. KM curves were constructed with stratified data based on quantiles 25th and 75th of gene expression. High group is represented in red and the low group in blue. The p-values correspond to the log-rank test with multiple test corrections employing the BH method.

**Figure S3**


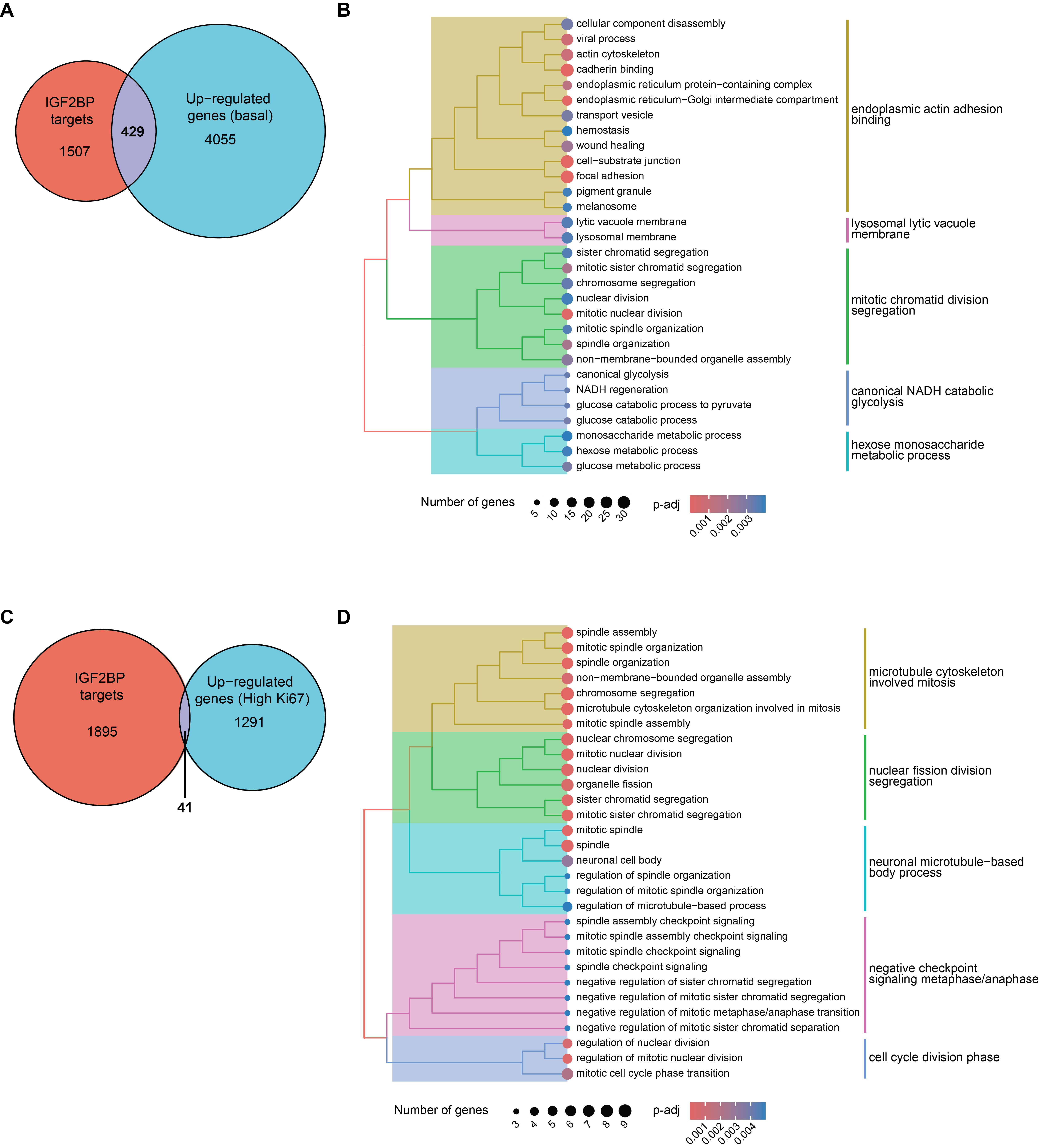


*Figure legend in the next page.*

**Supplementary Figure S3**. **Gene set enrichment analysis of IGF2BP targets.** (**A**) Overlap between eCLIP-derived IGF2BP1-3 targeted genes and upregulated genes in the basal subtype versus control. (**B**) Tree plot displaying the GO terms enriched in the upregulated IGF2BP targets from **A.** (**C**) Overlap between eCLIP-derived IGF2BP1-3 targets and upregulated genes in high Ki67 versus low Ki67. (**D**) Tree plot displaying the GO terms enriched in the upregulated IGF2BP targets from **C**.

**Figure S4**


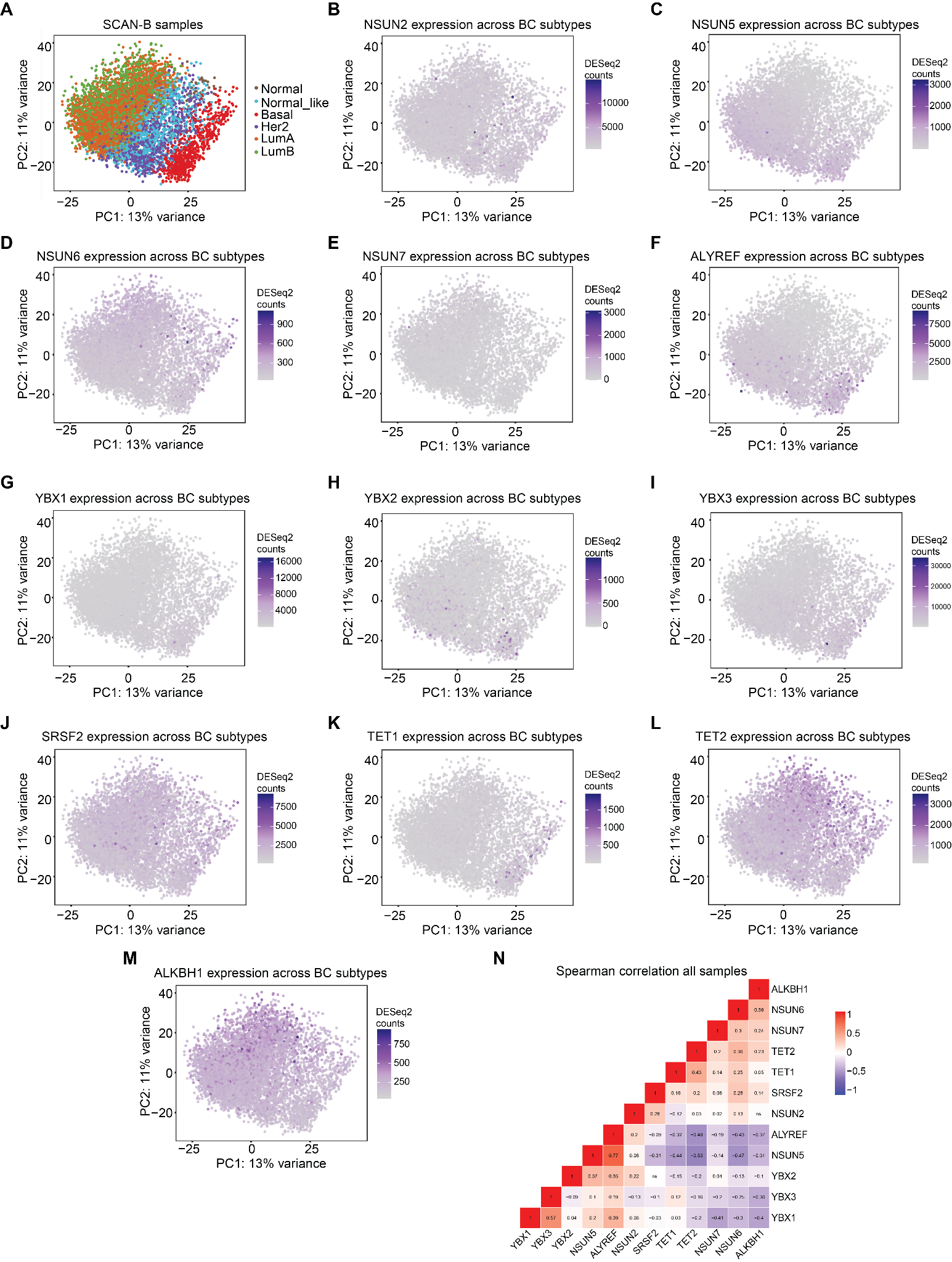


*Figure legend in the next page.*

**Supplementary Figure S4**. **PCA and Spearman correlation of the m^5^C mRMPs across SCAN-B samples, related to Figure 2.** (**A**) PCA with annotated subtypes. Each point on the scatter plot represents an individual sample. The color code corresponds to the breast cancer subtype. (**B-M**) PCA plots showing gene expression of m^5^C mRMPs, where each point represents a SCAN-B sample and the color gradient reflects the RNA expression level across samples (DESeq2-normalized counts). (**N**) Spearman correlation scores between m^5^C mRMPs using the full SCAN-B cohort. The color gradient ranges represent the degree of correlation and range from -1 (blue) to +1 (red).

**Figure S5**


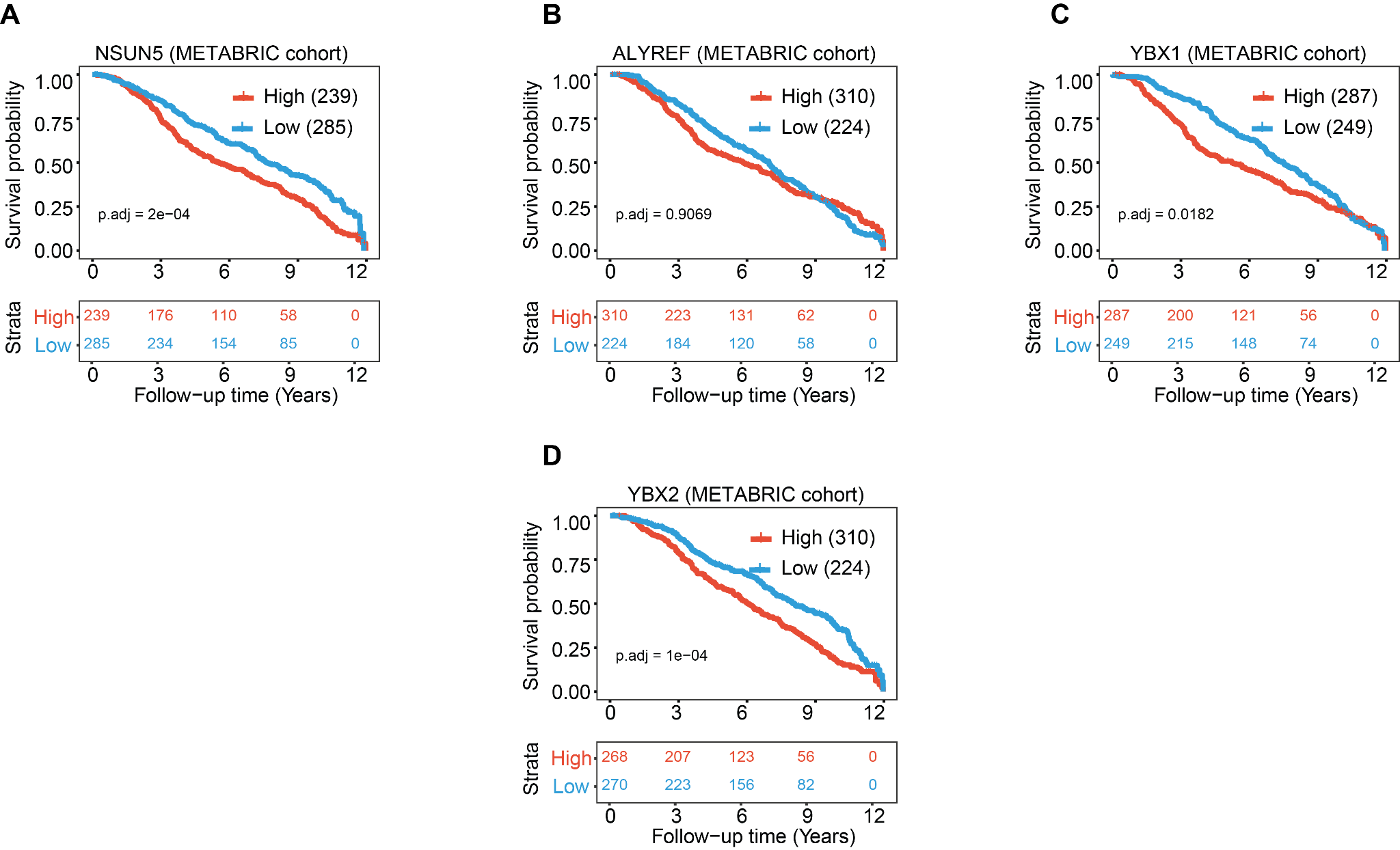


**Supplementary Figure S5**. **OS analysis of TNBC-overexpressed m^5^C mRMPs in METABRIC, related to Figure 2.** (**A-D**) OS analysis conducted in the METABRIC dataset for NSUN5, ALYREF, YBX1, and YBX2. KM curves were constructed with stratified data based on quantiles 25th and 75th of gene expression. High group is represented in red and the low group in blue. The p-values correspond to the log-rank test with multiple test corrections employing the BH method.

**Figure S6**


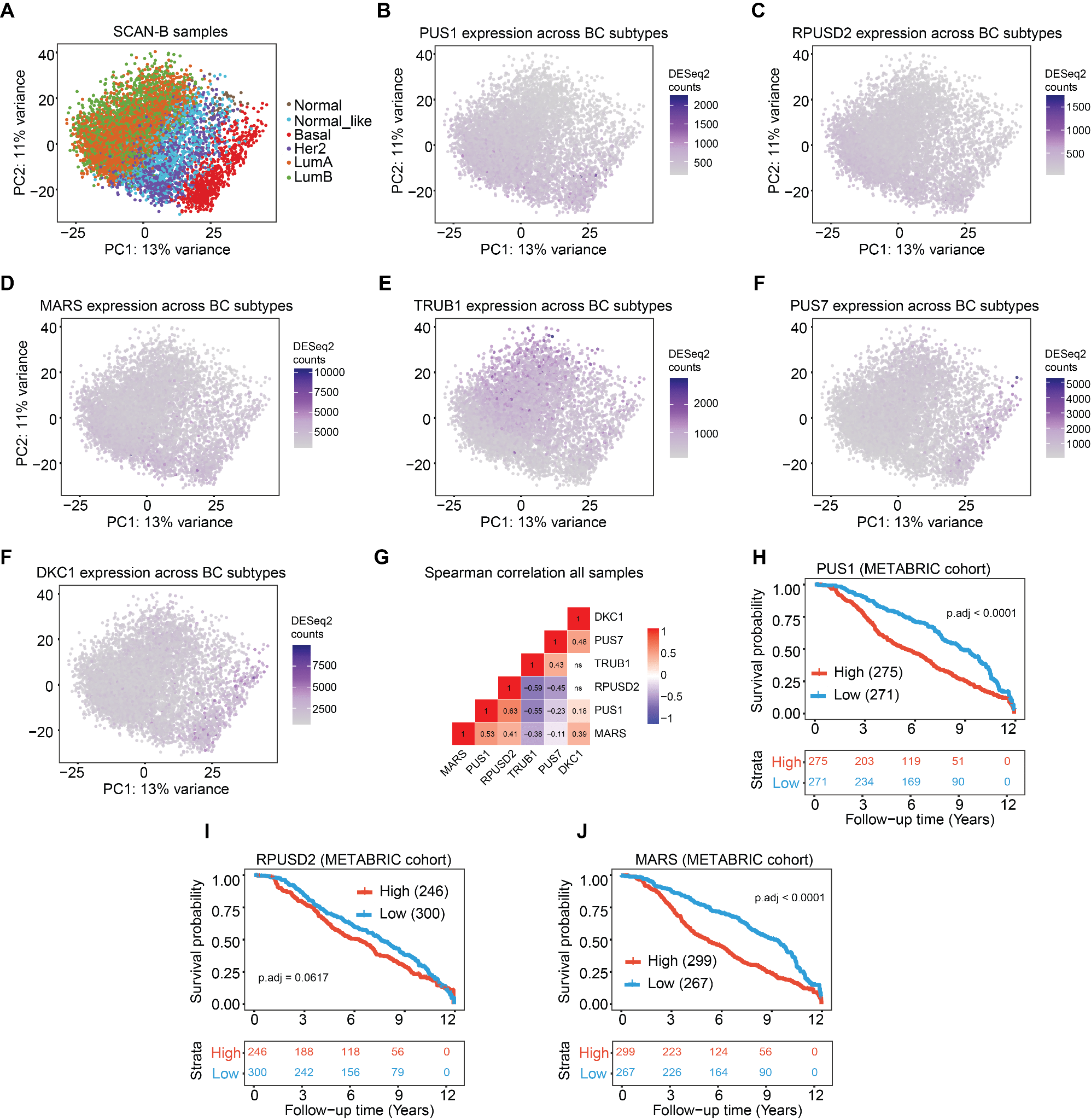


*Figure legend in the next page.*

**Supplementary Figure S6. PCA and Spearman correlation for Ψ-related mRMPs across SCAN-B cohort and OS analysis of METABRIC, related to Figure 3.** (**A**) PCA with annotated subtypes. Each point on the scatter plot represents an individual sample. The color code corresponds to the breast cancer subtype. (**B-F**) PCA plots showing gene expression of Ψ mRMPs, where each point represents a SCAN-B sample and the color gradient reflects the RNA expression level across samples (DESeq2-normalized counts). (**G**) Spearman correlation scores between Ψ mRMPs using the full SCAN-B cohort. The color gradient ranges represent the degree of correlation and range from -1 (blue) to +1 (red). (**H-J**) OS analysis conducted in the METABRIC dataset for Ψ mRMPs. KM curves were constructed with stratified data based on quantiles 25th and 75th of gene expression. High group is represented in red and the low group in blue. The p-values correspond to the log-rank test with multiple test corrections employing the BH method.

**Figure S7**


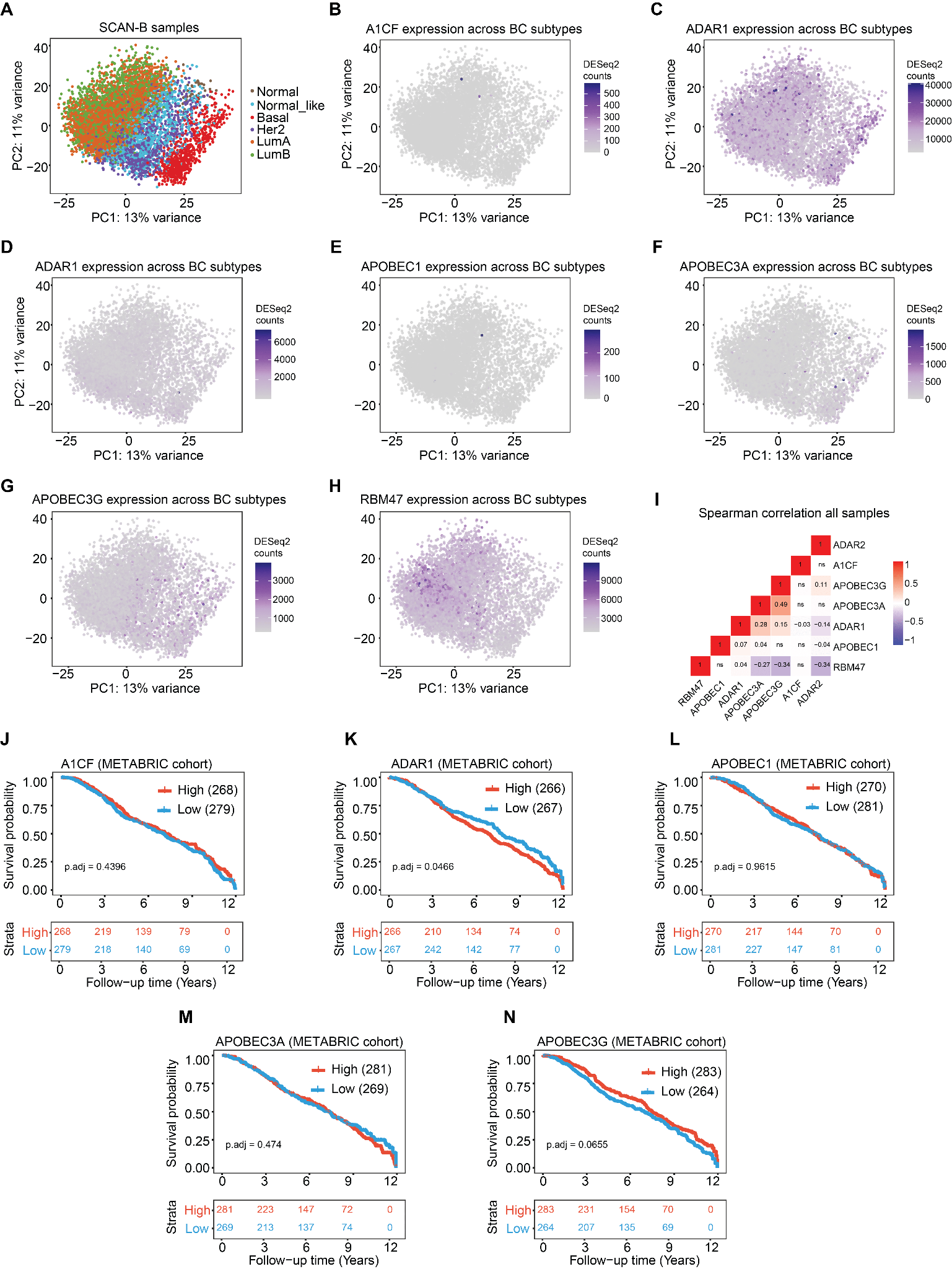


*Figure legend in the next page.*

**Supplementary Figure S7**. **PCA and Spearman correlation for RNA editing enzymes in SCAN-B cohort and OS analysis of METABRIC**, **related to Figure 4**.

(**A**) PCA with annotated subtypes. Each point on the scatter plot represents an individual sample. The color code corresponds to the breast cancer subtype. (**B-H**) PCA plots showing gene expression of RNA editing enzymes and cofactors, where each point represents a SCAN-B sample and the color gradient reflects the RNA expression level across samples (DESeq2-normalized counts). (**I**) Spearman correlation scores between RNA editing enzymes using the full SCAN-B cohort. The color gradient ranges represent the degree of correlation and range from -1 (blue) to +1 (red). (**J-N**) OS analysis conducted in the METABRIC dataset for RNA editing enzymes. KM curves were constructed with stratified data based on quantiles 25th and 75th of gene expression. High group is represented in red and the low group in blue. The p-values correspond to the log-rank test with multiple test corrections employing the BH method.

**Figure S8**


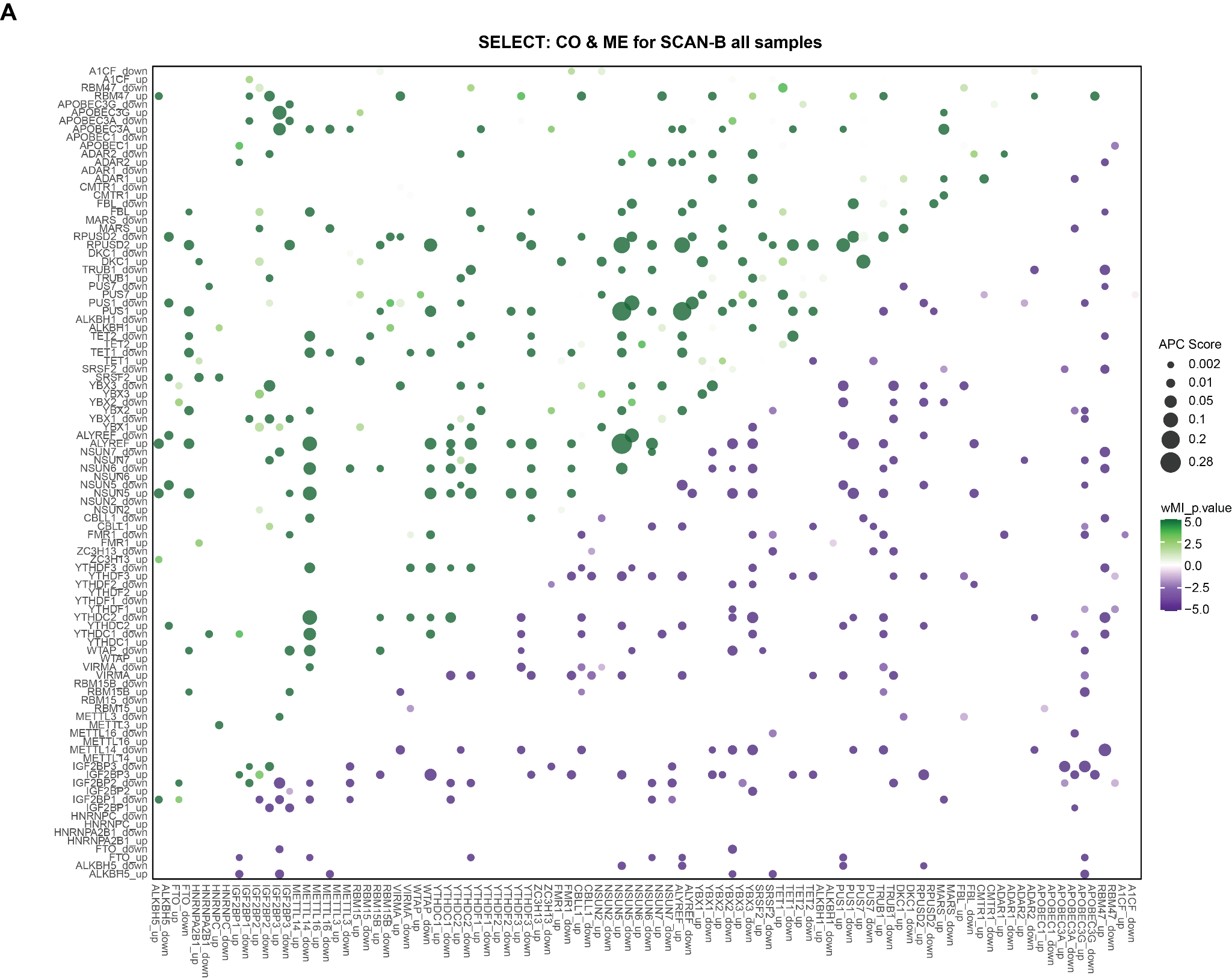


**Supplementary Figure S8**. **CO/ME analysis for all mRMPs across SCAN-B cohort, related to Figure 5.** Analysis of co-ocurrence (CO) and mutual exclusivity (ME) among the 49 mRMPs included in this study, employing the SELECT algorithm. The color gradient represents the weighted Mutual Information (wMI) p-value, reflecting the type of interaction, with violet representing mutual exclusivity (ME) and green indicating co-occurrence (CO). Bubble size corresponds to the Average Product Correction (APC) effect size.

**Figure S9**


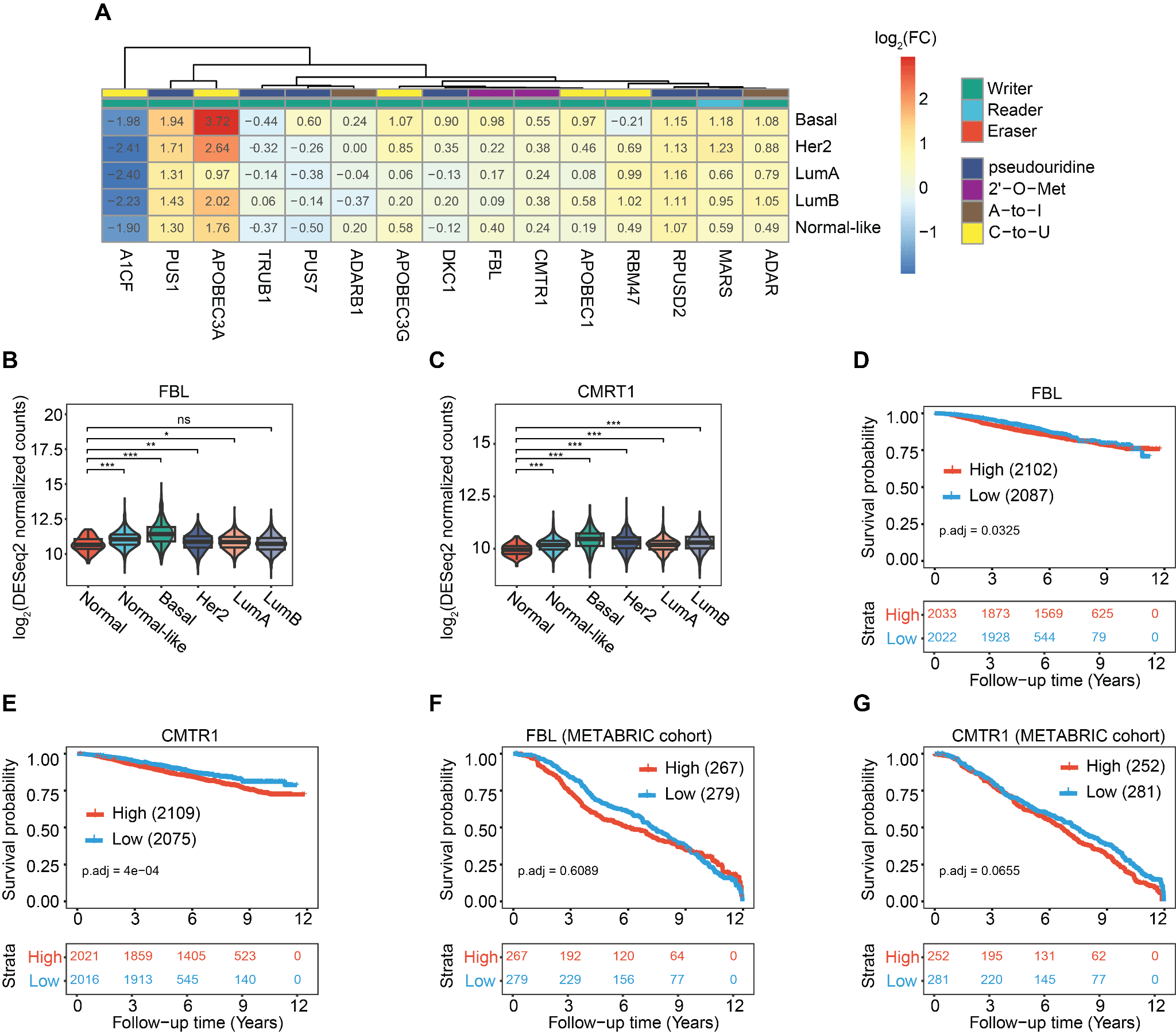


*Figure legend in the next page.*

**Supplementary Figure S9**. **Analysis of Fibrillarin and CMRT1 in the SCAN-B dataset.** (**A**) Heatmap displaying the log2(FC) values for the differential expression analysis of 2’O-Me, Ψ and RNA editing mRMPs across breast cancer subtypes against the cohort of the normal breast samples (control group). Rows represent the different subtype vs control comparisons and columns represent mRMPs. The cell value is the log2(FC) of gene expression between two conditions. RMPs highlighted in red were found up-regulated in the basal type. (**B,C**) RNA levels of FBL and CMRT1 across the different breast cancer subtypes. (**D,E**) OS analysis conducted in the SCAN-B dataset for FBL and CMRT1. KM curves were constructed with stratified data based on quantiles 25th and 75th of gene expression. High group is represented in red and the low group in blue. The p-values correspond to the log-rank test with multiple test corrections employing the BH method. (**F,G**) OS analysis conducted in the METABRIC dataset for FBL and CMRT1.

**Figure S10**


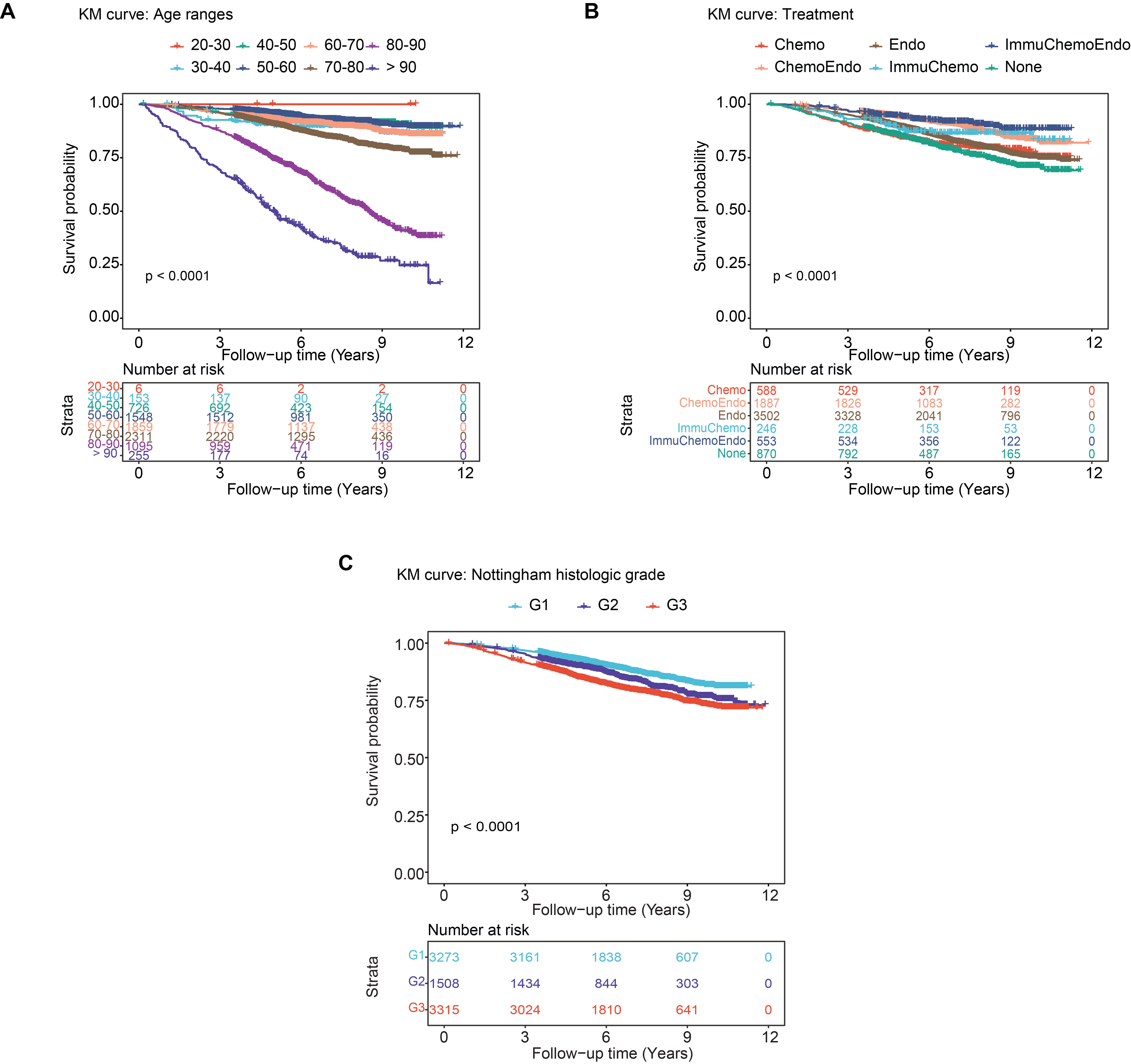


**Supplementary Figure S10**. **Overall survival analyses for relevant clinical variables of SCAN-B samples.** (**A**) KM curves spitted based on age groups ranging from 20 to more than 90. The x axis represents the time in years from the diagnosis to the last follow-up or death and y axis is the survival probability. (**B**) KM curves for different treatments and combinations of those to which the patients were subjected (Endo: endoscopic delivery, Chemo: chemotherapy, Immu: Immunotherapy). The x axis represents the time in years from the diagnosis to the last follow-up or death and y axis is the survival probability. (**C**) KM curves representing the three Nottingham histologic grades (G1, G2, and G3). The x axis represents the time in years from the diagnosis to the last follow-up or death and y axis is the survival probability.
